# Smoking status trajectories, intergenerational socioeconomic mobility and depression: Preliminary results from 107,734 French adults (18 to 75 years) of the CONSTANCES cohort

**DOI:** 10.1192/j.eurpsy.2023.1126

**Published:** 2023-07-19

**Authors:** A. J. Andersen, M. Mary-Krause, M. Melchior

**Affiliations:** 1 IPLESP, ERES, INSERM, Sorbonne University, Paris, France

## Abstract

**Introduction:**

Cigarette smoking prevalence is significantly higher among people with mental health problems than among the general population. Smoking accounts for much of the reduction in life expectancy associated with mental illness, why the high co-occurrence of smoking and mental health illness is a major public health concern. Persons belonging to socioeconomical disadvantaged groups have higher risk of mental health conditions and also higher smoking rates.

**Objectives:**

In this study we aim to examine smoking trajectories among adult smokers between 2012 and 2020. Furthermore, we aim to investigate differences in smoking trajectories by adult depression by taking into consideration participants intergenerational socioeconomic mobility (ISEM).

**Methods:**

Analyses were based on data from CONSTANCES, a French general population cohort conducted from 2012 to 2020. In total were 107,734 participants included after exclusion of never smokers. Depression was measured by the CES-D scale, and depression was classified with a score ≥16. ISEM is based on childhood (maternal and parental occupational grade) and adult socioeconomic position (SEP), and low ISEM includes those with low SEP as child and adult and high ISEM those with consistent high SEP. Group-based trajectories modelling (GBTM) was used to determine smoking status trajectories. To address the association between ISEM and smoking trajectory class we used multinomial logistic regression with former smokers as reference class adjusted for depression, household income, sex and age.

**Results:**

We identified five smoking trajectories 1) Former smokers (56.6%), 2) Long-term smokers (26.4%), 3) Intermediate smokers (3.3%), 4) Early quitters (5.0%) and 5) Late quitters (8.7%). Preliminary results from multinomial logistic regression showed that persons with low ISEM had higher odds of depression (OR [95%CI]=1.91 [1.77;2.06]) than those with high ISEM. Participants with low ISEM had higher odds of being long-term smoking than former smokers compared to those with high ISEM (ORa [95%CI]=1.55 [1.43;1.67]). Furthermore, those with low ISEM had lower odds of being in any of the other smoking trajectory groups vs. former smokers compared to those with high ISEM (ORa [95%CI]=0.82 [0.69;0.97]) for intermediate smokers, ORa [95%CI]=0.75 [0.66;0.85]) for early quitters, and ORa [95%CI]=0.78 [0.70;0.87]) for late quitters).

**Conclusions:**

Preliminary results showed an association between ISEM and smoking trajectories in our study. Persons with low ISEM are more likely to be long-term smokers. Future analysis should consider the effect of depression as a mediating factor on the association between ISEM and smoking trajectories.

**Disclosure of Interest:**

None Declared

